# Development of a new method for collecting hemolymph and measuring phenoloxidase activity in *Tribolium castaneum*

**DOI:** 10.1186/s13104-018-4041-y

**Published:** 2019-01-07

**Authors:** Hiroko Tabunoki, Neal T. Dittmer, Maureen J. Gorman, Michael R. Kanost

**Affiliations:** 1 Department of Science of Biological Production, Graduate School of Agriculture, Tokyo University of Agriculture and Technology, 3-5-8 Saiwai-cho, Fuchu, Tokyo 183-8509 Japan; 2 Department of Biochemistry and Molecular Biophysics, Kansas State University, 141 Chalmers Hall, Manhattan, KS 66506-3702 USA

**Keywords:** Phenoloxidase, Melanization, Hemolymph, Small insects, Innate immune response, Melanin, Dopamine, *Tribolium castaneum*

## Abstract

**Objective:**

Hemolymph plays many important roles in the physiology of an insect throughout its lifetime; however, for small-bodied insects, studies are lacking because of the difficulties encountered while collecting hemolymph. The objective of our study was to develop a method to collect hemolymph plasma from various stages of *Tribolium castaneum* and to evaluate phenoloxidase activity in the plasma samples. We first designed a procedure for easily and quickly collecting clear hemolymph plasma from *T. castaneum*.

**Results:**

By using this method, we collected approximately 5 µl plasma from 30 individuals at the larval, pupal or adult stages. And then, we studied the expression of phenoloxidase by performing western blot analysis of the plasma samples and found that phenoloxidase is present in hemolymph in each developmental stage. We also measured phenoloxidase activity in control plasma and plasma treated with Gram-positive bacteria, *Micrococcus luteus*. Phenoloxidase activity was greater in some of the *M. luteus*-treated plasma samples compared with control samples. Thus, we developed a method to collect hemolymph plasma that is suitable for studies of phenoloxidase activity.

**Electronic supplementary material:**

The online version of this article (10.1186/s13104-018-4041-y) contains supplementary material, which is available to authorized users.

## Introduction

Insect hemolymph plays many important roles in the physiology of an insect, such as transporting nutrients and hormones to cells and tissues, maintaining the correct moisture ratio and optimal body temperature, maintaining body shape, and protecting the insect from pathogens [1]. In addition, some protein components in hemolymph plasma can differ between females and males throughout the developmental process and thus play specific roles in females and males. By investigating the biochemical components of hemolymph, the insect species can be better characterized. In the case of a honeybee, methods for collecting hemolymph were sought, and antennae method for hemolymph sampling (AMHS) is propounded as the quickest and most reliable method for collecting hemolymph [2, 3]. However, for many insects the collection and analysis of hemolymph has been difficult because of their tiny body size relative to the honeybee, and more methods need to be developed to resolve this issue [4–6].

The red flour beetle (*Tribolium castaneum*) is a model insect of the order Coleoptera. The genome of this species is well characterized, and methods to induce systemic RNA interference (RNAi) are available [7–10]. We were interested in understanding the immune response system in *T. castaneum* hemolymph; however, *T. castaneum* is a small-size insect, and collection of enough hemolymph for biochemical assays has been difficult.

Phenoloxidase (PO) is one of the plasma proteins in hemolymph [11]. It is expressed as an inactive zymogen, and is activated through specific proteolytic cleavage in response to wounding or infection [12]. Activated phenoloxidase hydroxylates tyrosine and oxidizes DOPA, dopamine, and dopamine derivatives, and subsequent chemical reactions lead to the formation of melanin [9]. Melanin participates in wound healing and immune-related melanization [13, 14]. Because we are interested in melanization, one of the goals of this study was to develop a plasma collection process that is compatible with phenoloxidase activity assays.

Here, we report a new method to easily and quickly collect clear hemolymph plasma from larval, pupal, and adult *T. castaneum*. Importantly, this method is compatible with analyses of phenoloxidase activity.

## Main text

### Methods

#### Insects

The *T. castaneum* GA-1 strain was used in all experiments. The insects were reared on whole-wheat flour containing 5% brewer’s yeast [15]. All insects were kept at 30 °C with a 16-h light/8-h dark cycle. We used last instar larvae which were selected using No. 25 sieve (Fisher scientific Inc., Waltham, MA, USA), pupae (0–4-day-old pupa), and adults (0–7-day-old) in this study.

#### Bioinformatics

The signal peptide sequences were analyzed using the SignalP 4.1 [16], and the molecular weights for PPO I (GenBank AY884063) and PPO II (GenBank AY884064) were predicted using Genetyx 13.0 (Genetyx Co. Ltd., Tokyo, Japan).

#### Hemolymph collection from *T. castaneum*

**Fig. 1:**
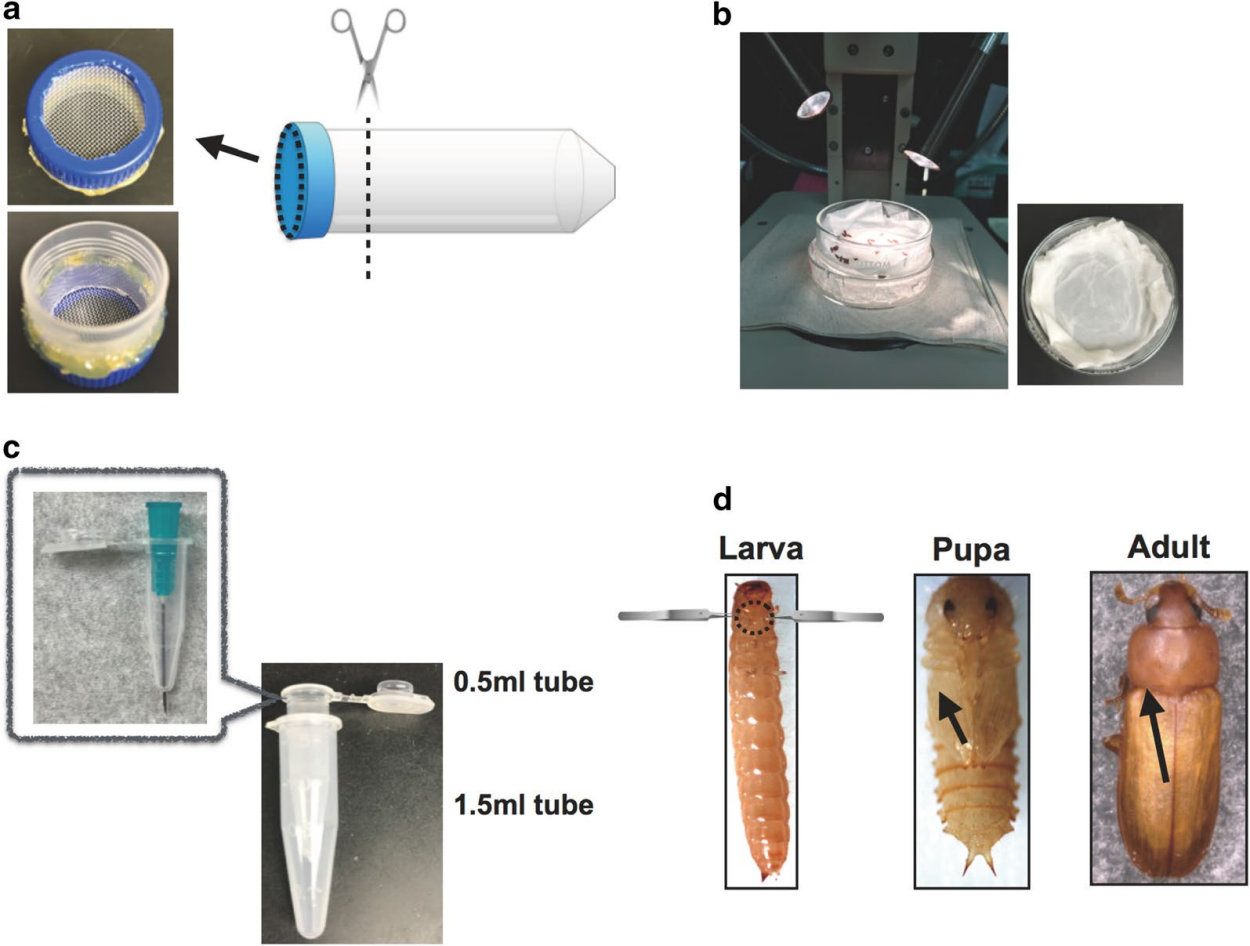
Method for collecting hemolymph. **a** Creating a handmade sieve for washing *T. castaneum.* The 50 ml tube drawing (http://g86.dbcls.jp/~togoriv/) is licensed under the HYPERLINK http://creativecommons.org/licenses/by/4.0/deed.ja. Creative commons display 4.0 license http://creativecommons.org/licenses/by/4.0/deed.ja. **b** Set up of stereomicroscope. **c** Structure of handmade collection tube, which consisted of a 0.5 ml tube with a hole at the bottom placed inside a 1.5 ml tube. **d** Location of punctures created in the larvae, pupae, and adults. Larvae were pinched at the root of their legs using two forceps, and the legs were pulled in opposite directions. The black broken circle indicates the location for creating a small tear. The forceps drawings (http://g86.dbcls.jp/~togoriv/) are licensed under the HYPERLINK http://creativecommons.org/licenses/by/4.0/deed.ja Creative commons display 4.0 license http://creativecommons.org/licenses/by/4.0/deed.ja. Pupae and adults were pierced with a needle at the points indicated by the black arrow. All protocols for collecting hemolymph from *T. castaneum* were performed on ice under a stereomicroscope

*Tribolium castaneum* larvae, pupae, and adults were counted (n = 30) and transferred to a small handmade sieve (Fig. 1a). The sieve was made by attaching a Nitex Nylon screen 630 µm (Thermo Fisher Scientific, Inc., Waltham, MA, USA) to part of a 50 ml plastic centrifuge tube (Corning Co. Ltd., One Riverfront Plaza, NY, USA). The larvae, pupae, and adults were washed with distilled water containing 0.1% Tween, then washed with tap water for 1 min, and finally rinsed with 70% ethanol. Moisture was removed using a paper towel. The insects were then transferred to a glass dish containing a slightly moist and flat paper tissue on the bottom. The glass dish was kept on ice to anesthetize the insects (Fig. 1b). To prepare for collecting the hemolymph plasma, a hole was punctured in a 0.5-ml tube using a 23-gauge needle (Thermo Fisher Scientific, Inc.,) (Fig. 1c). To separate the males from the females, the sex of the pupal and adult insects was determined according to USDA Beetle Wrangling Tips, Sexing Tribolium [17] (Additional file 1: Figure S1).

To collect hemolymph from the larvae, pupae, and adults, *T. castaneum* individuals were handled as shown in Fig. 1d. The larvae were grasped at the base of their legs using two forceps (Fig. 1d, larva), and the legs were pulled in opposite directions to create a tear in the cuticle between them, with care to avoid damaging the gut. Pupae were pierced through the anterior region of an elytron (which in the pupa is on the ventral surface) using a 28 gauge needle (BD 1/2 cc U-100 Insulin Syringe #329461) (Fig. 1d, pupa indicated by a black arrow). The adults were pierced on the dorsal side, between the pronotum and elytron (Fig. 1d, adult black arrow). The insects were then transferred to the ice-cold collection tubes (Fig. 1c). The collection tubes were centrifuged using the 5418 centrifuge (Eppendorf Inc. Co. Ltd. Hamburg, Germany) at 12,000×*g* for 10 min at 4 °C, and the supernatant, which we refer to as plasma, was transferred to a new tube. For some experiments, this protocol was modified to prevent hemolymph melanization. For experiments measuring phenoloxidase activity, 20 μl bleeding buffer (100 mM trisodium citrate, 17.8 mM citric acid, pH 5.5) was placed at the bottom of the 1.5 ml collection tubes. For samples used in immunodetection, 2 mg phenylthiourea (Sigma-Aldrich, St. Louis, MO, USA) was added to the 1.5 ml tube.

#### Measurement of phenoloxidase activity

The plasma collected using bleeding buffer was used to measure phenoloxidase activity. Two microliters of plasma were added to a 1.5-ml tube containing 1 μl filter-sterilized deionized water (control) or 1 μl *Micrococcus luteus* (1 μg/μl suspension, Sigma-Aldrich), and the tubes were incubated for 10 min at room temperature (RT). After incubation, these plasma samples were transferred to separate wells of a 96-well plate, after which 4 μl of a 50 mM dopamine hydrochloride solution in 1 mM HCl (Sigma-Aldrich), and 93 μl of 20 mM 3-(*N*-morpholino)propanesulfonic acid (MOPS) (pH 7.0) containing 25 mM calcium chloride (Sigma-Aldrich) were added to each well. The absorbance at 470 nm was monitored once/minute for 1 h at 30 °C using a microplate spectrophotometer (Synergy HT, BioTek co. ltd., Winooski, VT USA). The reaction curves started with a lag phase followed by a linear phase. Linear regression was used to calculate the changes in absorbance per minute during the linear phase of each reaction. One unit of phenoloxidase activity was defined as ΔA_470_ = 0.001/min [18]. The assay was performed in triplicate. Statistical significance was determined by two-tailed Student’s t-test using Excel (Microsoft, Redmond, WA, USA). p values of < 0.05 were considered to be significant.

#### Western blotting

Plasma samples (0.5 μl) containing phenylthiourea were heated in SDS-PAGE sample buffer and then separated by electrophoresis on 4–12% BisTris gels with MOPS running buffer (Thermo Fisher Scientific, Inc.) under reducing conditions according to the manufacturer’s instructions. The proteins were then transferred to nitrocellulose membranes (Bio-Rad Laboratories, Inc., Hercules, CA, USA) using the method described by Towbin et al. [19]. The membranes were incubated in blocking buffer composed of 3% milk and Tris-buffered saline (TBS) at pH 7.4, including 0.1% Tween 20 (TBS-T) for 1 h at RT, incubated in rabbit anti-phenoloxidase polyclonal antiserum (KSU37) as the primary antibody (diluted 1:2000) in blocking buffer for 1 h, and washed three times with TBS-T for 5 min. The polyclonal antiserum KSU37 used for immunoblot analysis detects both the PPOI and PPOII subunits of *Manduda sexta* prophenoloxidase [20]. The washed membranes were incubated with goat anti-rabbit immunoglobulin (Ig)G (H+L) alkaline phosphatase (AP) conjugate 1:3000 (Bio-Rad Laboratories, Inc.) in blocking buffer for 1 h at RT and then washed twice with TBS-T for 5 min and with TBS for 5 min. The membranes were developed using an AP Conjugate Substrate Kit (Bio-Rad Laboratories, Inc.) for 6 min.

### Results and discussion

In this study, we developed methods for easily and quickly collecting clear plasma from *T. castaneum* and assessed the presence and activity of phenoloxidase in the plasma. First, we collected approximately 5 µl of hemolymph from 30 individuals at each developmental stage (Additional file 1: Table S1).

**Fig. 2:**
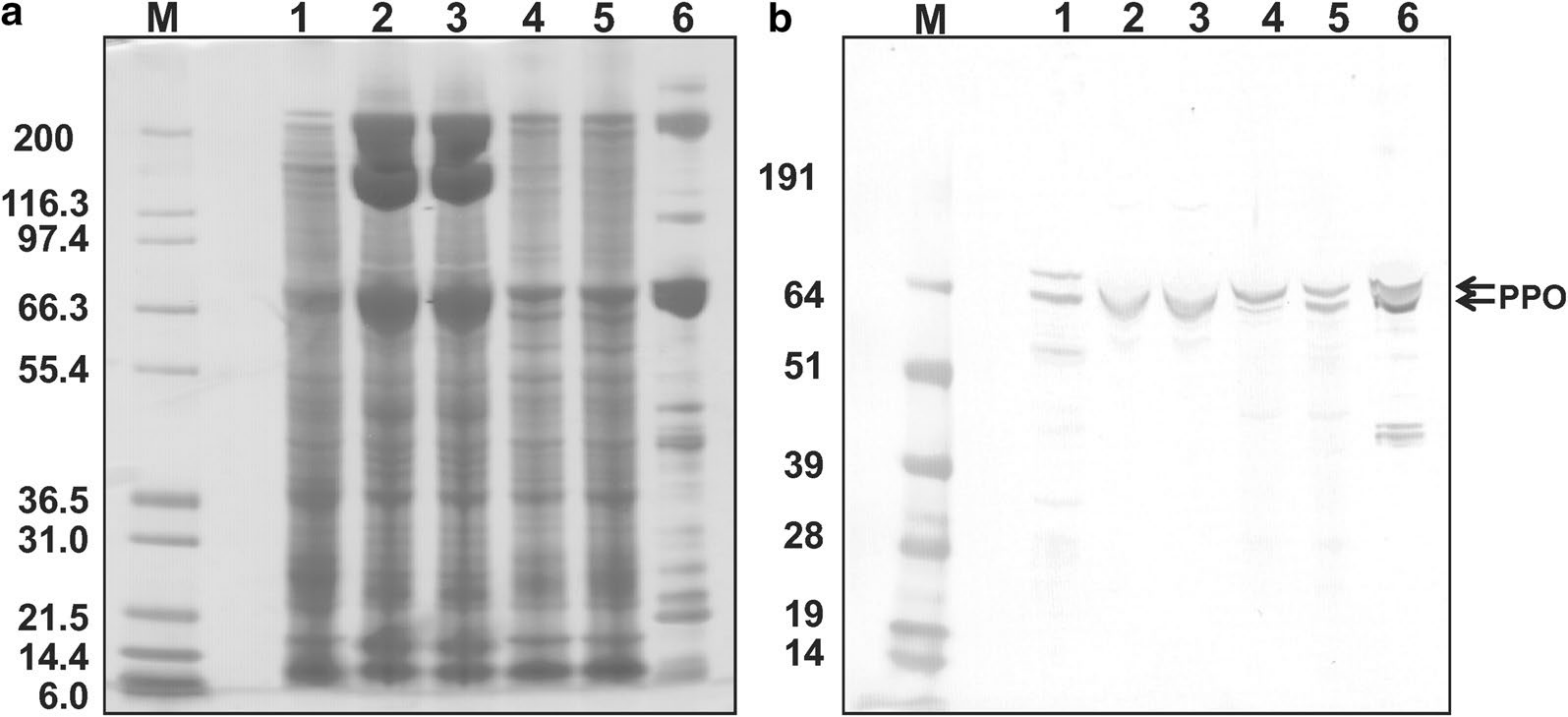
Dectection of phenoloxidase in larvae, pupae and adults. Plasma protein samples (0.5 µl) from *T. castaneum* larva, lane 1; male pupa, lane 2; female pupa, lane 3; male adult, lane 4; and female adult, lane 5; *M. sexta* larval plasma, lane 6 (as a positive control) were separated by SDS-PAGE, transferred to nitrocellulose, and probed with anti-*M. sexta* phenoloxidase antibody. **a** Coomassie blue staining of the separated proteins. **b** Immunoblotting. Lane M represents molecular weight markers. Arrows indicate bands of the expected size for prophenoloxidase detected by prophenoloxidase antiserum

*Tribolium castaneum* expresses two phenoloxidase genes: PPOI and PPOII [21]. PPOI has a predicted molecular weight of 79,099 Da and PPOII has a predicted molecular weight of 79,338 Da. We examined the utility of available antisera against *M. sexta* phenoloxidase to identify phenoloxidase in *T. castaneum* plasma. *M. sexta* anti-phenoloxidase antibody reacted with one or two polypeptides of approximately 80 kDa in all *T. castaneum* plasma samples (Fig. 2b). Because phenoloxidase bands of the expected size were detected, we conclude that the plasma samples were not significantly contaminated with gut contents (which contain proteolytic enzymes) (Fig. 2a). Also, we found that collected hemolymph plasma may include some cellular proteins because our samples contained many more protein bands than plasma from *Tenebrio molitor,* which is a larger Coleopteran insect than *T. castaneum* [22].

It has been reported that neither PPO mRNA was expressed from the late pupa stage to the early adult stage [21]. However, phenoloxidase was found in adult plasma in this study. These results suggest that *T. castaneum* phenoloxidase is a stable protein, persisting from the pupal stage into the adult stage.

We attempted to measure phenoloxidase activity in *T. castaneum* plasma by monitoring the oxidation of the substrate dopamine according to a previously described phenoloxidase assay [18]. However, this method was not suitable for *T. castaneum* plasma because of rapid hemolymph melanization that occurred in the larval and adult samples before the assay could be performed.

To circumvent this problem, we tested several bleeding and assay buffers to determine which combinations would best suppress oxidation of endogenous catechols during the collection process but give reliable results during the activity assay; these buffer combinations are shown in Additional file 1: Table S2. Buffer combinations 1 to 3 were not suitable for measuring phenoloxidase activity as the change in absorbance did not progress linearly with time (Additional file 1: Figures S2, S3, S4, Table S3).

**Fig. 3:**
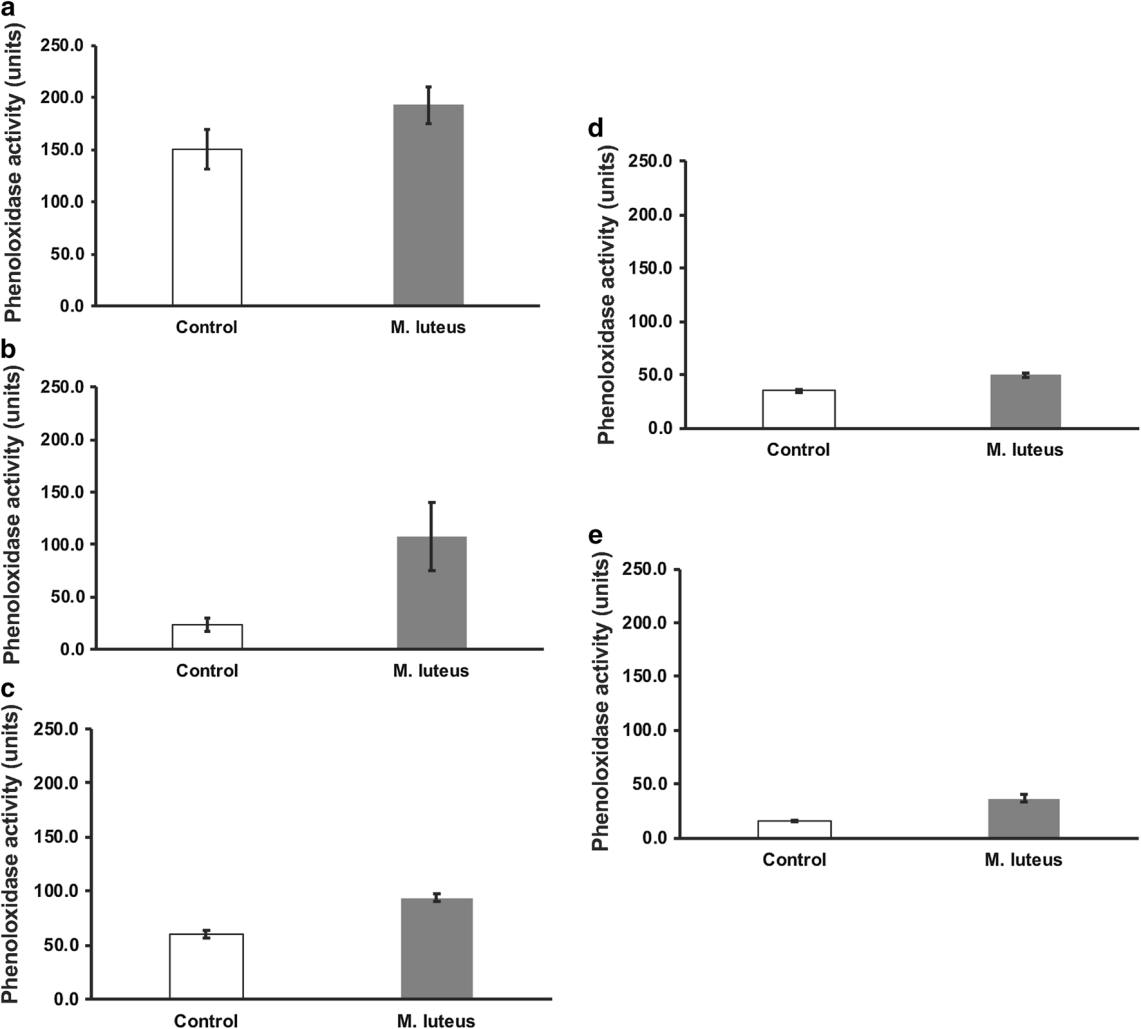
Phenoloxidase activity of plasma samples from *T. castaneum*. Control, no added bacteria; *M. luteus* indicates plasma samples treated with *M. luteus* prior to PO assay. **a** Larva (p = 0.183), **b** male pupa (p = 0.065), **c** female pupa (p = 0.003), **d** male adult (p = 0.008), and **e** female adult (p = 0.007). Error bars indicate standard errors (n = 3)

The assay was slightly improved using buffer combination 4 (Additional file 1: Figure S5, Table S3), as this prevented premature melanization in the larval plasma but not the adult plasma. Previous reports for *Tenebrio molitor*, *Alphitobius diaperinus*, and *Hermetia illucens* indicated that oxidation of endogenous dopamine was inhibited using a bleeding buffer at pH 4.0; however, phenoloxidase activity was highest at approximately pH 6.0 in these species [23]. Thus, we assessed additional buffer combinations according to our previous findings. Buffer combination 5 completely blocked early plasma melanization, however phenoloxidase activity was very low in all samples (Additional file 1: Figure S6, Table S3). Finally, we found that buffer combination 6, with collecting hemolymph into citrate buffer at pH 5.5, was suitable for minimizing melanization during hemolymph collection while still allowing for sufficient dopamine oxidation for measuring phenoloxidase activity (Additional file 1: Figure S7, Fig. 3).

For some studies of insect immunity, phenoloxidase in hemolymph or plasma is intentionally activated by the addition of an immune elicitor. To verify that the plasma collection method we developed is suitable for these kinds of studies, we observed whether phenoloxidase activity was higher after incubating the plasma with Gram-positive bacteria, *M. luteus*. For these experiments, we used buffer combination 6. We found that phenoloxidase activity increased after treatment with *M. luteus* in plasma samples from female pupae and male and female adults, but the increase observed in plasma from larvae and male pupae was not statistically significant (Fig. 3). It is possible that the phenoloxidase in those samples was already partially activated by the collection process.

### Conclusion

We developed an easy method to collect hemolymph plasma from *T. castaneum*. Our methods allow for the conducting of experiments which have previously been challenging due to the difficulty in collecting sufficient amounts of hemolymph from this insect. Also, we were able to clarify that prophenoloxidase protein is a stable protein, persisting from the pupal stage into the adult stage of the *T. castaneum* hemolymph. This new method could be helpful for various analyses of *T. castaneum* hemolymph, for example, biochemical assays, other enzyme assays, proteome analysis, metabolomic analysis, and tracking of labeled substances or proteins in the hemolymph in *T. castaneum*.

## Limitations

This method may not be suitable for studying hemocytes as they are removed and possibly damaged during the centrifugation step. This method may also damage other cells whose contents may then contaminate the plasma sample.

## Additional file

**Additional file 1: Figure S1.** Identifying the sex of pupal and adult *T. castaneum.* The structure of the genital papillae is markedly different between female and male pupae. The pupae were sexed by examining the structure of the genital papillae (on the last abdominal segment), which are markedly different between female and male pupae (indicated with black arrow). For adults, sex was determined by the presence (male) or absence (female) of a patch of bristles (“sex patch”; indicated by white arrow) on the femur of the prothoracic legs. **Figure S2.** Observation of the reaction curve in experimental condition 1. The absorbance (A470) was monitored, and calculated as mO.D./min (ΔA470 = 0.001/min), and plotted on the graph. Control, no added bacteria; *M. luteus* indicates plasma samples treated with *M. luteus* prior to PO assay. A; larva control, B; larva *M. luteus*, C; Adult control, D; Adult *M. luteus*. **Figure S3.** Observation of the reaction curve in Experimental condition 2. The absorbance (A470) was monitored, and calculated as mO.D./min (ΔA470 = 0.001/min), and plotted on the graph. Control, no added bacteria; *M. luteus* indicates plasma samples treated with *M. luteus* prior to PO assay. A; larva control, B; larva *M. luteus*, C; Adult control, D; Adult *M. luteus*. **Figure S4.** Observation of the reaction curve in Experimental condition 3. The absorbance (A470) was monitored, and calculated as mO.D./min (ΔA470 = 0.001/min), and plotted on the graph. Control, no added bacteria; *M. luteus* indicates plasma samples treated with *M. luteus* prior to PO assay. A; larva control, B; larva *M. luteus*, C; Adult control, D; Adult *M. luteus*. **Figure S5.** Observation of the reaction curve in Experimental condition 4. The absorbance (A470) was monitored, and calculated as mO.D./min (ΔA470 = 0.001/min), and plotted on the graph. Control, no added bacteria; *M. luteus* indicates plasma samples treated with *M. luteus* prior to PO assay. A; larva control, B; larva *M. luteus*, C; Adult control, D; Adult *M. luteus*. **Figure S6.** Observation of the reaction curve in Experimental condition 5. The absorbance (A470) was monitored, and calculated as mO.D./min (ΔA470 = 0.001/min), and plotted on the graph. Control, no added bacteria; *M. luteus* indicates plasma samples treated with *M. luteus* prior to PO assay. A; larva control, B; larva *M. luteus*, C; Adult control, D; Adult *M. luteus*. **Figure S7.** Observation of the reaction curve in Experimental condition 6. The absorbance (A_470_) was monitored, and calculated as mO.D./min (ΔA470 = 0.001/min), and plotted on the graph. Control, no added bacteria; *M. luteus* indicates plasma samples treated with *M. luteus* prior to PO assay. A; larva control, B; larva M. luteus, C; Pupa-male control, D; Pupa-male *M. luteus*, E; Pupa-female control, F; Pupa-female *M. luteus*, G; Adult-male control, H; Adult-male *M. luteus*, I; Adult-female control, J; Adult-female *M. luteus*. **Table S1.** Plasma collected from different developmental stages (n = 30). **Table S2.** Assay buffer combinations tested for measuring phenoloxidase (PO) activity. **Table S3.** Phenoloxidase activity in experimental combination 1 to 5.

## Abbreviations

AMHS: antennae method for hemolymph sampling
RNAi: RNA interference
PO: phenoloxidase
