# Supplementary material for: Development of a new method for collecting hemolymph and measuring phenoloxidase activity in Tribolium castaneum
Source: BMC Res Notes. 2019 Jan 7;12:7. doi: 10.1186/s13104-018-4041-y (PMC6323729; doi:10.1186/s13104-018-4041-y)
Supplement: Supplementary file 1 — Additional file 1: Figure S1. Identifying the sex of pupal and adult T. castaneum. The structure of the genital papillae is markedly different between female and male pupae. The pupae were sexed by examining the structure of the genital papillae (on the last abdominal segment), which are markedly different between female and male pupae (indicated with black arrow). For adults, sex was determined by the presence (male) or absence (female) of a patch of bristles (“sex patch”; indicated by white arrow) on the femur of the prothoracic legs. Figure S2. Observation of the reaction curve in experimental condition 1. The absorbance (A470) was monitored, and calculated as mO.D./min (ΔA470 = 0.001/min), and plotted on the graph. Control, no added bacteria; M. luteus indicates plasma samples treated with M. luteus prior to PO assay. A; larva control, B; larva M. luteus, C; Adult control, D; Adult M. luteus. Figure S3. Observation of the reaction curve in Experimental condition 2. The absorbance (A470) was monitored, and calculated as mO.D./min (ΔA470 = 0.001/min), and plotted on the graph. Control, no added bacteria; M. luteus indicates plasma samples treated with M. luteus prior to PO assay. A; larva control, B; larva M. luteus, C; Adult control, D; Adult M. luteus. Figure S4. Observation of the reaction curve in Experimental condition 3. The absorbance (A470) was monitored, and calculated as mO.D./min (ΔA470 = 0.001/min), and plotted on the graph. Control, no added bacteria; M. luteus indicates plasma samples treated with M. luteus prior to PO assay. A; larva control, B; larva M. luteus, C; Adult control, D; Adult M. luteus. Figure S5. Observation of the reaction curve in Experimental condition 4. The absorbance (A470) was monitored, and calculated as mO.D./min (ΔA470 = 0.001/min), and plotted on the graph. Control, no added bacteria; M. luteus indicates plasma samples treated with M. luteus prior to PO assay. A; larva control, B; larva M. luteus, C; Adult control, D; Adult [file 13104_2018_4041_MOESM1_ESM.docx]

**Development of a new method for collecting hemolymph and measuring phenoloxidase activity in *Tribolium castaneum***

Hiroko Tabunoki^1,2, *^, Neal T. Dittmer^2^, Maureen J. Gorman^2^ and Michael R. Kanost^2^

^1^ Department of Science of Biological Production, Graduate School of Agriculture, Tokyo University of Agriculture and Technology, 3-5-8 Saiwai-cho, Fuchu, Tokyo 183-8509, Japan.

^2^Department of Biochemistry and Molecular Biophysics, Kansas State University, 141 Chalmers Hall, Manhattan, KS 66506-3702, USA

*To whom correspondence should be addressed. E-mail:h_tabuno@cc.tuat.ac.jp

Additional Figures

**Additional figure S1. Identifying the sex of pupal and adult** ***T. castaneum***

The structure of the genital papillae is markedly different between female and male pupae. The pupae were sexed by examining the structure of the genital papillae (on the last abdominal segment), which are markedly different between female and male pupae (indicated with black arrow). For adults, sex was determined by the presence (male) or absence (female) of a patch of bristles ("sex patch"; indicated by white arrow) on the femur of the prothoracic legs.


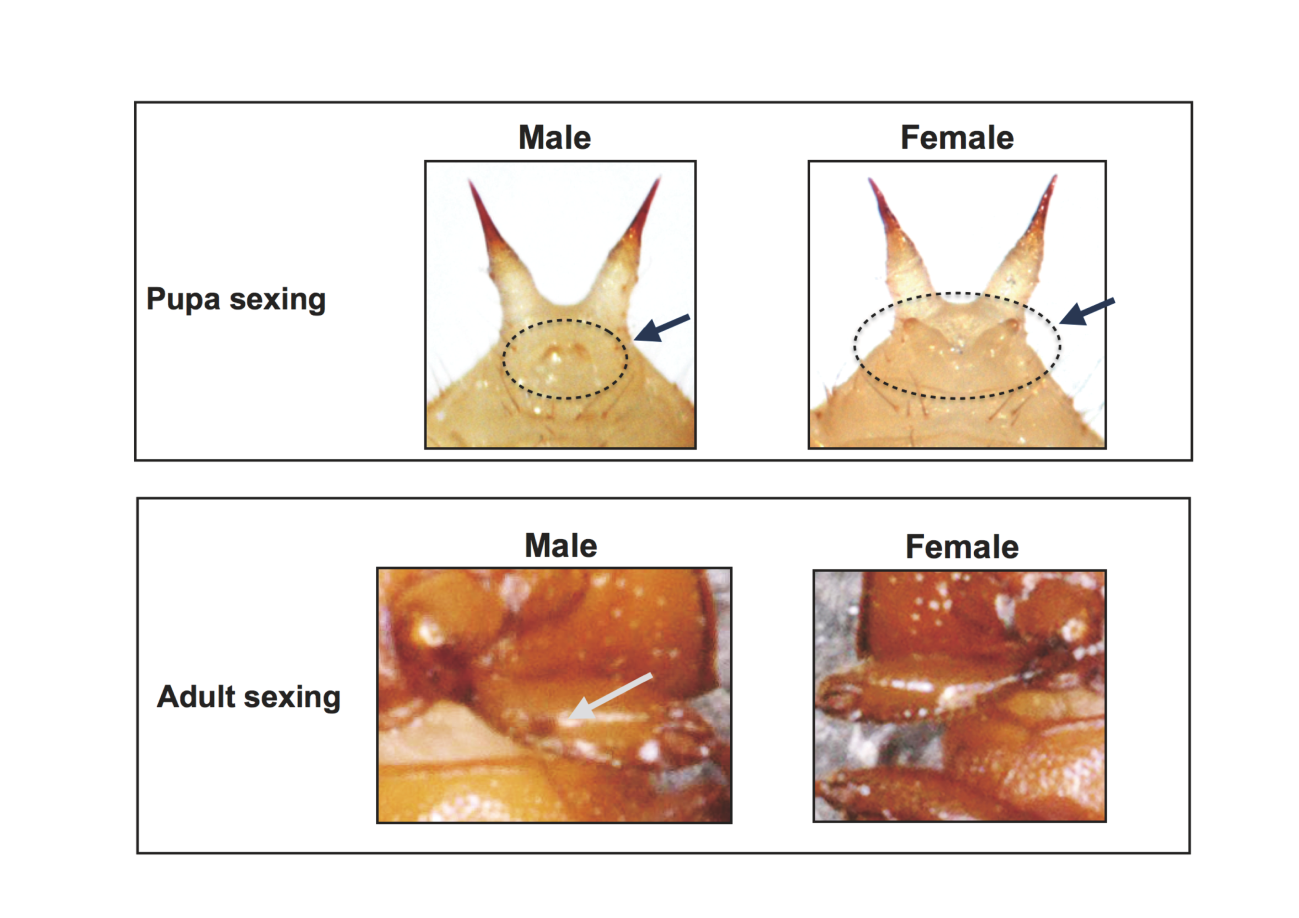


**Additional figure S2. Observation of the reaction curve in** **Experimental condition 1**

The absorbance (A_470_) was monitored, and calculated as mO.D./min (ΔA470 = 0.001/min), and plotted on the graph. Control, no added bacteria; *M. luteus* indicates plasma samples treated with *M. luteus* prior to PO assay. A; larva control, B; larva M. luteus, C; Adult control, D; Adult *M. luteus*.


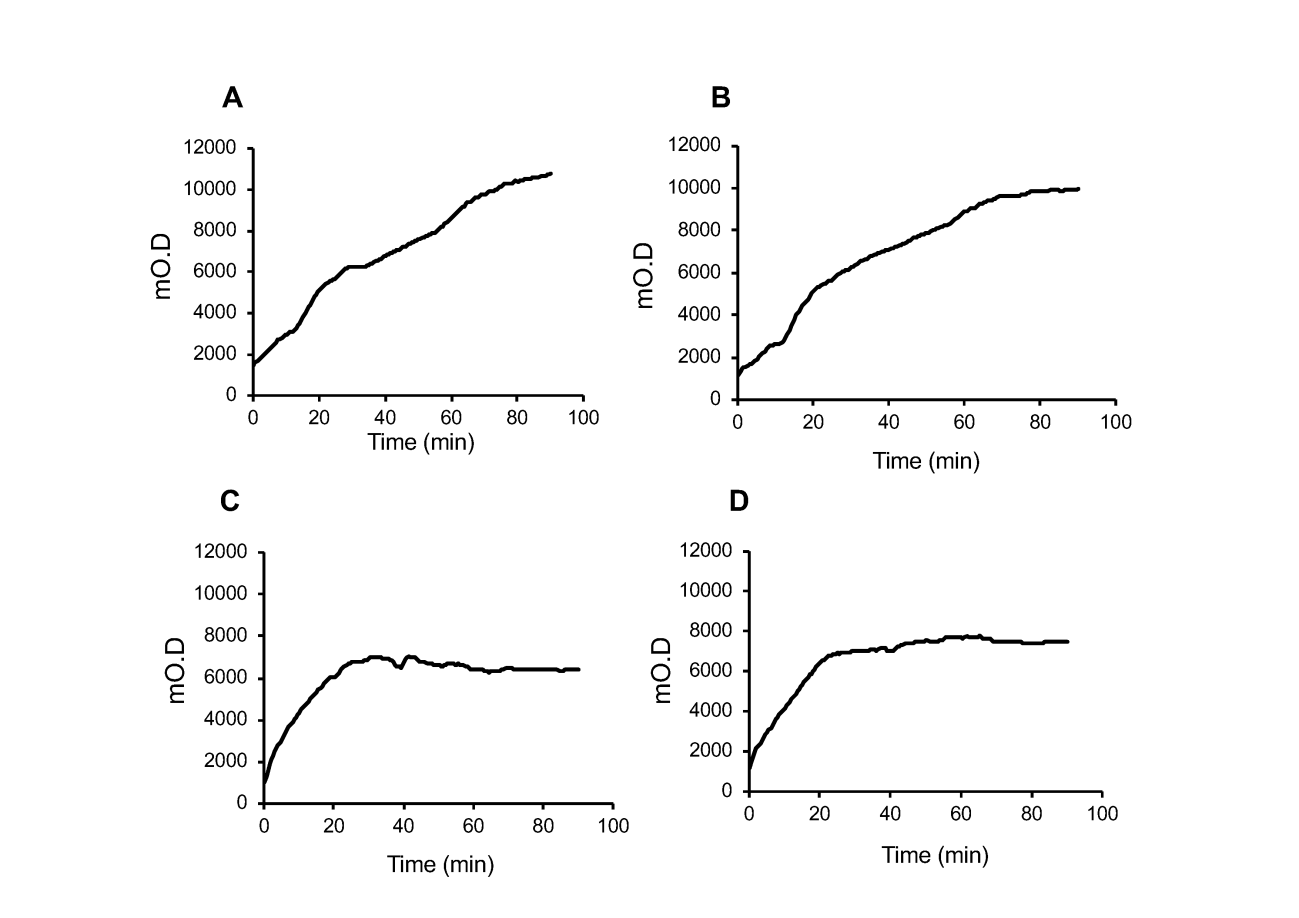


**Additional figure S3. Observation of the reaction curve in** **Experimental condition 2**

The absorbance (A_470_) was monitored, and calculated as mO.D./min (ΔA470 = 0.001/min), and plotted on the graph. Control, no added bacteria; *M. luteus* indicates plasma samples treated with *M. luteus* prior to PO assay. A; larva control, B; larva M. luteus, C; Adult control, D; Adult *M. luteus*.


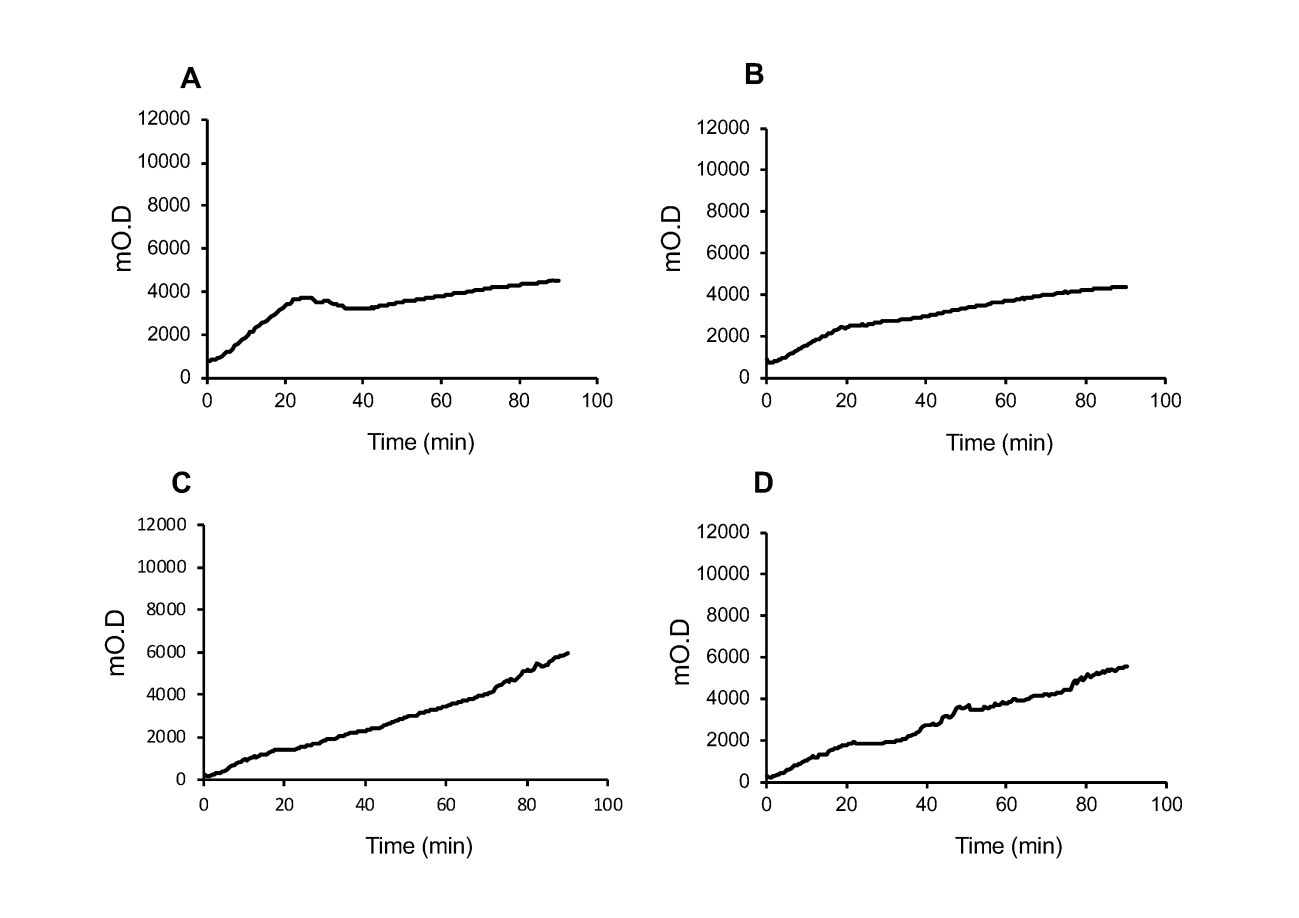


**Additional figure S4. Observation of the reaction curve in** **Experimental condition 3**

The absorbance (A_470_) was monitored, and calculated as mO.D./min (ΔA470 = 0.001/min), and plotted on the graph. Control, no added bacteria; *M. luteus* indicates plasma samples treated with *M. luteus* prior to PO assay. A; larva control, B; larva M. luteus, C; Adult control, D; Adult *M. luteus*.


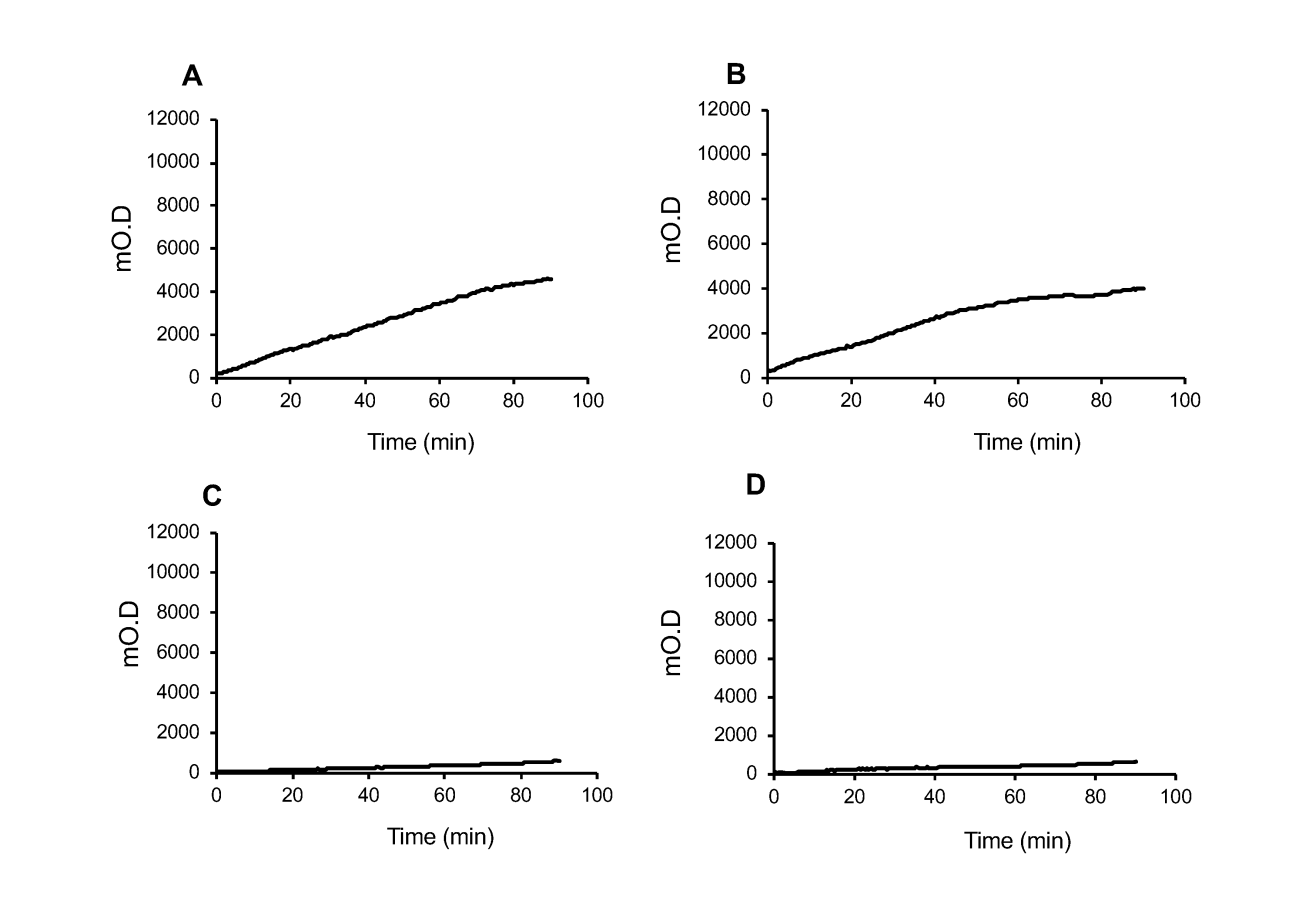


**Additional figure S5. Observation of the reaction curve in** **Experimental condition 4**

The absorbance (A_470_) was monitored, and calculated as mO.D./min (ΔA470 = 0.001/min), and plotted on the graph. Control, no added bacteria; *M. luteus* indicates plasma samples treated with *M. luteus* prior to PO assay. A; larva control, B; larva M. luteus, C; Pupa control, D; Pupa *M. luteus*, E; Adult control, F; Adult *M. luteus*.


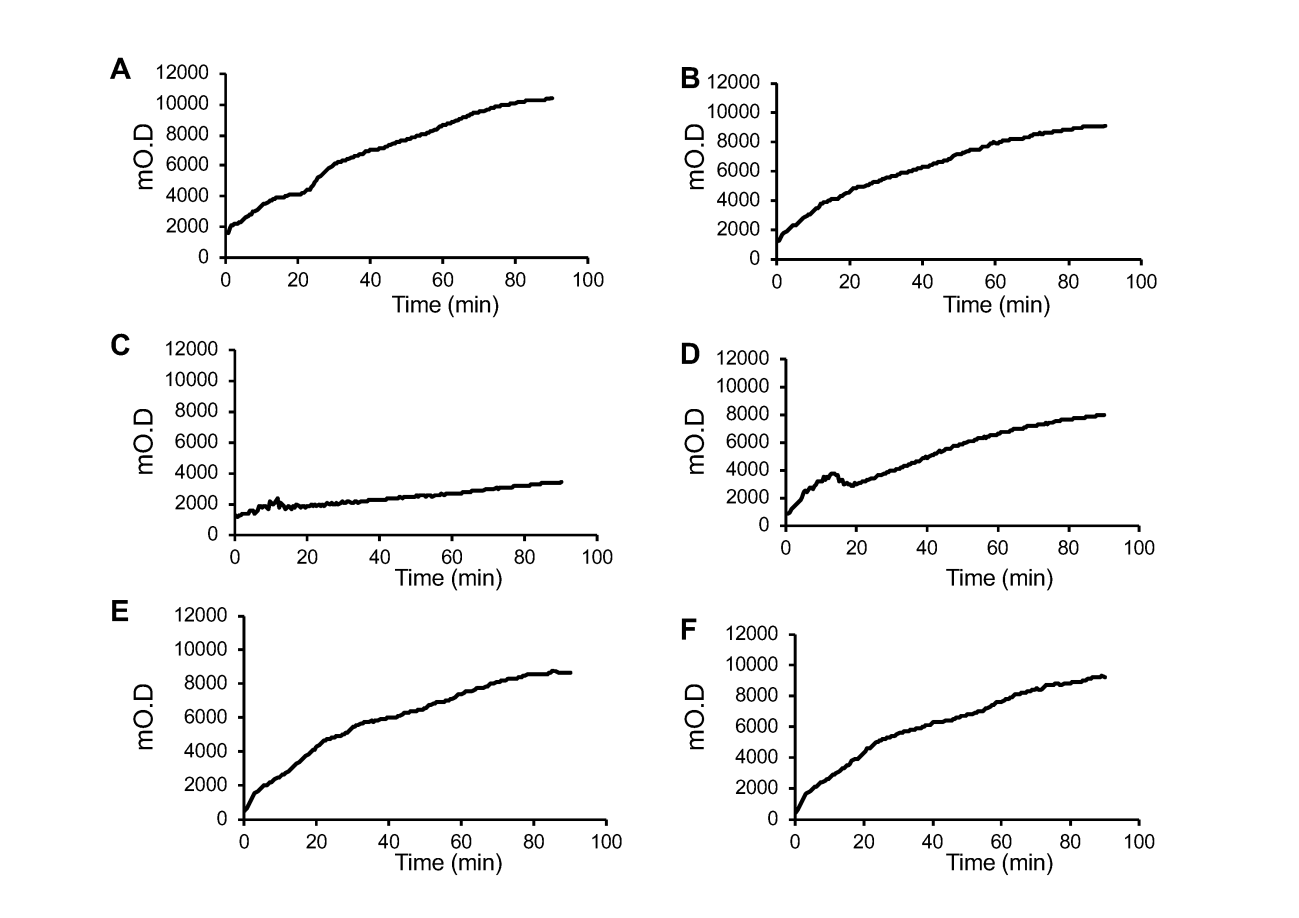


**Additional figure S6. Observation of the reaction curve in** **Experimental condition 5**

The absorbance (A_470_) was monitored, and calculated as mO.D./min (ΔA470 = 0.001/min), and plotted on the graph. Control, no added bacteria; *M. luteus* indicates plasma samples treated with *M. luteus* prior to PO assay. A; larva control, B; larva M. luteus, C; Pupa control, D; Pupa *M. luteus*, E; Adult control, F; Adult *M. luteus*.


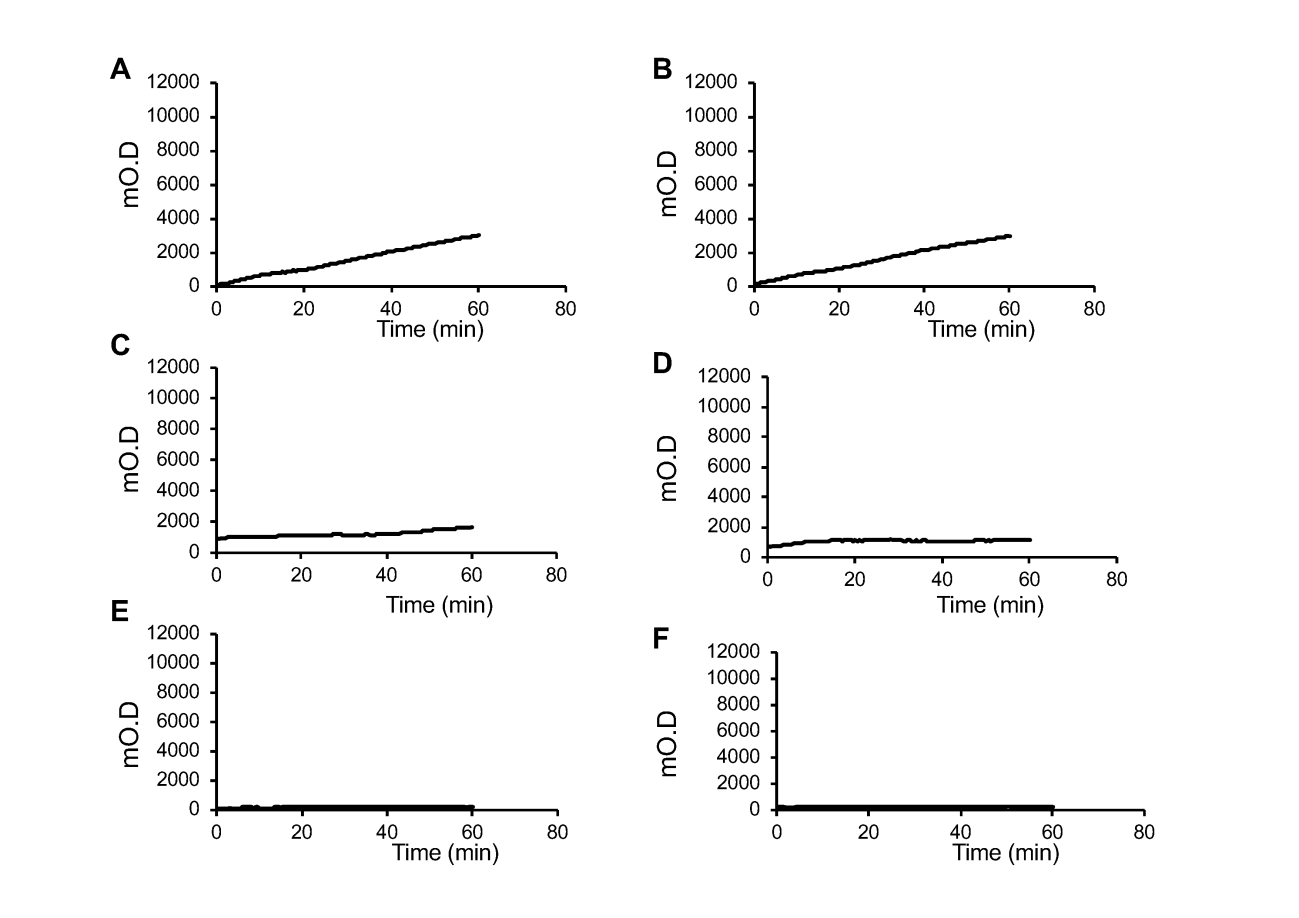


**Additional figure S7. Observation of the reaction curve in** **Experimental condition 6**

The absorbance (A_470_) was monitored, and calculated as mO.D./min (ΔA470 = 0.001/min), and plotted on the graph. Control, no added bacteria; *M. luteus* indicates plasma samples treated with *M. luteus* prior to PO assay. A; larva control, B; larva M. luteus, C; Pupa-male control, D; Pupa-male *M. luteus*, E; Pupa-female control, F; Pupa-female *M. luteus*, G; Adult-male control, H; Adult-male *M. luteus*, I; Adult-female control, J; Adult-female *M. luteus*.


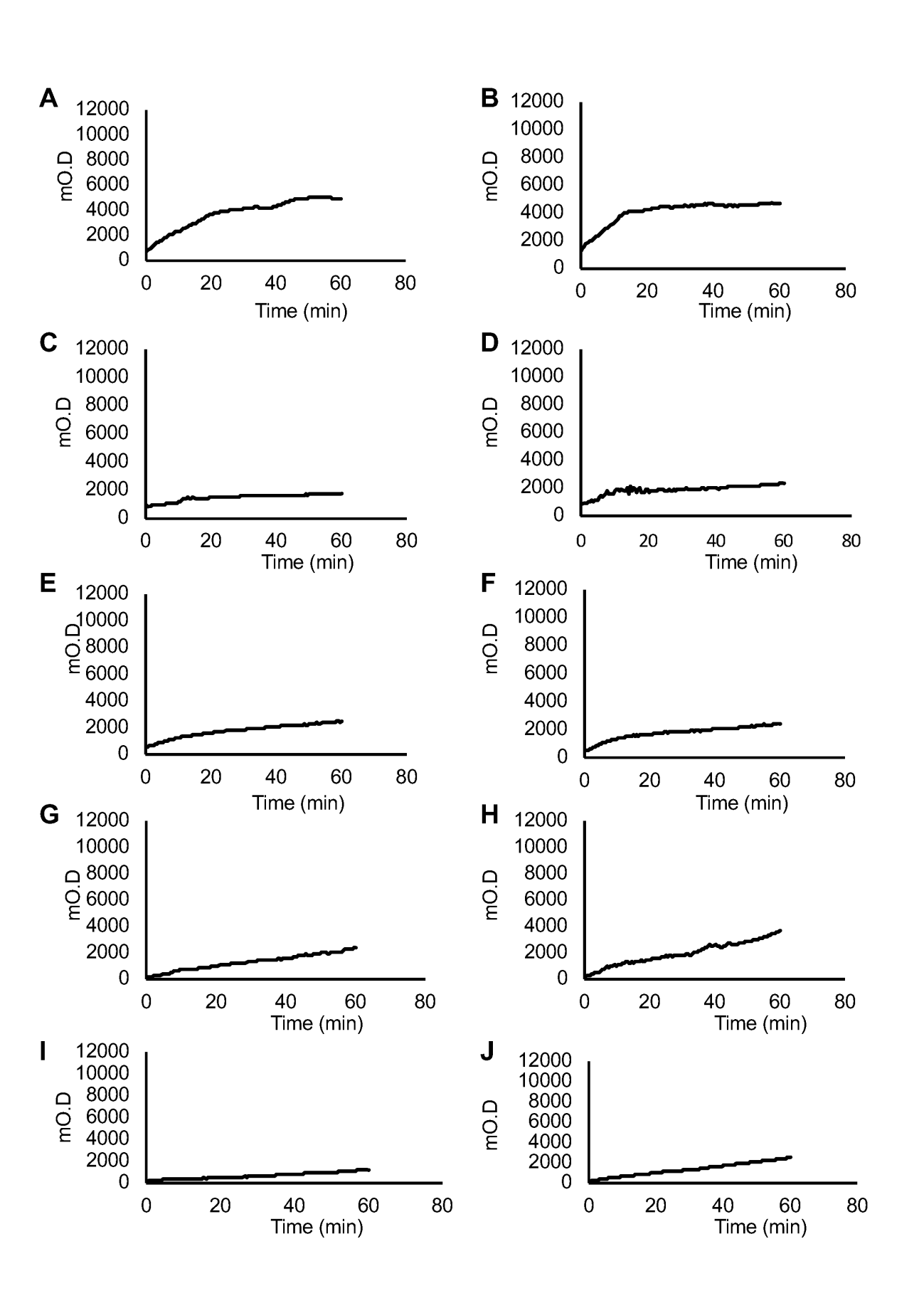


Additional Tables

**Additional Table S1. Plasma collected from different developmental stages (n = 30).**

| Developmental stage | Yield (mean ± SD) μl | Plasma per individual (μl) |
| --- | --- | --- |
| Larva | 4.75 ± 0.5 | 0.16/larva |
| Pupa | 5 ± 0 | 0.17/pupa |
| Adult | 5.7 ± 6.1 | 0.19/adult |

**Additional Table S2. Assay buffer combinations tested for measuring phenoloxidase (PO) activity.**

| experimental  condition | Bleeding buffer | Assay buffer | Assay temperature (°C) | Reference |
| --- | --- | --- | --- | --- |
| 1 | 50 mM ascorbic acid/50 mM Phosphate buffer pH 6.5 | 50 mM Posphate buffer pH 6.5, 2 mM dopamine | 37 | Bleeding buffer [1,2] Incubation temperature [3] |
| 2 | 136 mM trisodium citrate, 26 mM citric acid, 20 mM EDTA, 15 mM NaCl pH 5.3 | 20 mMTris-HCl pH 8.0, 10 mM CaCl_2_, 2 mM dopamine | 30 | Assay Methods for *T. molitor* [4] |
| 3 | 136 mM trisodium citrate, 26 mM Citric acid, 20 mM EDTA, 15 mM NaCl pH 5.3 | 20 mM MOPS pH 7.0, 10 mM CaCl_2_, 2 mM dopamine | 30 | Modify Assay Methods for *T. molitor* [4] |
| 4 | 96 mM trisodium citrate, 4 mM Citric acid pH 6.5 | 20 mM MOPS pH 7.0, 10 mM CaCl_2_, 2 mM dopamine | 30 |  |
| 5 | 100 mM trisodium citrate, 56.2 mM Citric acid pH4.5 | 20 mM MOPS pH7.0, 25 mM CaCl_2_, 2 mM dopamine | 30 |  |
| 6 | 100 mM trisodium citrate, 17.8 mM Citric acid pH5.5 | 20 mM MOPS pH 7.0, 25 mM CaCl_2_, 2 mM dopamine | 30 |  |

**Additional Table S3. Phenoloxidase activity in experimental combination 1 to 5.**

| Experimental number | Larva control | Larva M. luteus | Pupa control | Pupa M. luteus | Adult control | Adult M. luteus |
| --- | --- | --- | --- | --- | --- | --- |
| 1 | 175.4 | 188.5 | N.M. | N.M. | 233.7 | 246.9 |
| 2 | 30.9 | 37.1 | N.M. | N.M. | 59.8 | 56.8 |
| 3 | 51.4 | 42.0 | N.M. | N.M. | 5.7 | 5.5 |
| 4 | 110.5 | 150.7 | 22.0 | 100.0 | 158.3 | 153.3 |
| 5 | 48.9 | 47.9 | 10.2 | 4.0 | 0.6 | 1.0 |

Phenoloxidase activity (units) was calculated as the mean value (n=3). “N.M.” indicates “not measure”.

**References**

1.Yamamura I. Yonekura M. Katsura Y. Ishiguro M. Funatsu M., Purification and some physico-chemical properties of phenoloxidase from the larvae of Housefly. Agric. Biol. Chem., 1980; 44: 55-59.

2.Yonekura M., Shimoda T., Funatsu M., Reinvestigation of purification of phenoloxidaze from larvae of Housefly. Agric. Biol. Chem., 1981; 45: 101-104.

3.Joop G, Roth O, Schmid-Hempel P, Kurtz J., Experimental evolution of external immune defences in the red flour beetle. J Evol Biol. 2014; 27(8): 1562-1571.

4.Lee KM., Lee KY., Choi HW., Cho MY., Kwon TH., Kawabata S., et al. Activated phenoloxidase from *Tenebrio molitor* larvae enhances the synthesis of melanin by using a vitellogenin-like protein in the presence of dopamine. Eur. J. Biochem. 2000; 267: 3695-3703
